# Safety of percutaneous microwave ablation under local anesthesia for uterine fibroids and adenomyosis

**DOI:** 10.1186/s13244-025-02149-5

**Published:** 2025-12-02

**Authors:** Ruyue Tian, Yahui Ma, Xuedi Han, Yufeng Wang, Jiajun Wang, Ya Sun, Nan Zhou, Yuqing Huang, XiaoHong Sun, Xin Zhang, Yandong Deng, Lei Liang

**Affiliations:** 1 Department of Ultrasound, Aerospace Center Hospital, No. 15 Yuquan Road, Haidian District, Beijing, China; 2 Department of Obstetrics and Gynecology, YiXian Hospital, No. 38 Changan Road, YiXian District, Baoding, China; 3 Department of Obstetrics and Gynecology, Emergency General Hospital, No. 29, Xibahe Nanli Road, Chaoyang District, Beijing, China; 4 Department of Ultrasound, The First Hospital of Hebei Medical University, 89 Donggang Road, Shijiazhuang, China

**Keywords:** Microwave ablation, Uterine fibroids, Adenomyosis, Adverse events, Minimally invasive surgery

## Abstract

**Objective:**

This study explored the incidence of adverse events (AEs) following microwave ablation (MWA) under local anesthesia and analyzed factors related to benign uterine diseases, including uterine fibroids (UFs) and adenomyosis (AM).

**Materials and methods:**

Overall, 366 patients who underwent percutaneous MWA were finally included in this study. Univariate and multivariate logistic regression analyses were performed to identify the main factors affecting AEs.

**Results:**

The overall AEs rate for benign uterine disease was 77.32% (283/366), and was significantly higher in patients with AM than in those with UFs (95.38% vs. 73.42%, *p* < 0.001). AM (odds ratio (OR) = 3.77, *p* = 0.039) and higher transformed symptom severity score (higher tSSS) (25–40: OR = 2.98, *p* = 0.007; > 40: OR = 2.36, *p* = 0.022) were independent risk factors for AEs. In the subgroup analysis of patients with UFs, moderate-to-severe pain during MWA was significantly associated with AE occurrence (OR = 2.35, *p* = 0.048) and abdominal pain (OR = 3.63, *p* < 0.001). Although multivariate regression analysis showed that higher tSSS (25–40: OR = 3.22, *p *= 0.003; > 40: OR = 3.32, *p* = 0.001) was an independent influencing factor for vaginal discharge, univariate analysis suggested that vaginal discharge risk also increased with FIGO 0–3 (OR = 2.53, *p* = 0.010).

**Conclusion:**

Our results demonstrated that AM and higher tSSS were identified as significant independent risk factors, facilitating better patient selection and improved patient counseling. Moderate-to-severe pain during MWA was strongly associated with AE occurrence, highlighting the need for further investigation of anesthesia optimization. Further, patients with FIGO 0–3 fibroids exhibited a higher risk of postoperative vaginal discharge, necessitating procedural refinement to preserve endometrial integrity.

**Critical relevance statement:**

Our study makes a significant contribution to the literature because it provides a comprehensive analysis of microwave ablation-related adverse events and their associated risk factors, facilitating better patient selection, procedural refinements, and improved patient counseling.

**Key Points:**

- This study addresses a critical gap in the literature by investigating the safety of ultrasound-guided microwave ablation (MWA) for uterine fibroids (UFs) and adenomyosis (AM) under local anesthesia.
- Our results demonstrated the overall AE rate for UFs and AM following MWA was 77.32%, with AM and higher transformed symptom severity scores identified as significant independent risk factors.
- Given the differences in AE risk between UFs and AM, as well as related risk factors, tailored treatment protocols should be considered to optimize outcomes.

**Graphical Abstract:**

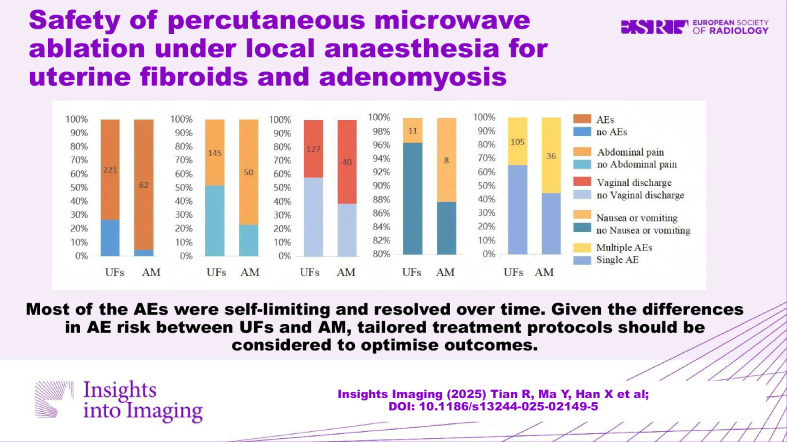

## Introduction

Uterine fibroids (UFs) and adenomyosis (AM) are common benign uterine diseases in women of reproductive age, and both are associated with estrogen. However, the pathophysiology, anatomical distributions, and clinical manifestations vary significantly between these conditions. Typical symptoms, such as abnormal uterine bleeding, progressive dysmenorrhea, uterine enlargement, and infertility, can substantially affect a patient’s quality of life [1, 2]. The current treatment options primarily consist of myomectomy or hysterectomy, which are performed using various surgical approaches. Although these procedures generally effectively alleviate symptoms, they may also result in varying degrees of fertility impairment [3]. Given the invasive nature and prolonged recovery associated with surgical interventions, minimally invasive surgery has gained acceptance among physicians and patients.

Ultrasound-guided microwave ablation (MWA) is a promising minimally invasive alternative, offering high therapeutic efficacy, a low incidence of adverse events (AEs), and a shorter post-treatment recovery period [4, 5]. Moreover, MWA enables real-time monitoring of the ablation target and surrounding tissues, allowing precise energy delivery to induce thermal destruction of pathological tissues while preserving adjacent structures. MWA has demonstrated satisfactory therapeutic effects on short-term symptom improvement and long-term volume reduction ratios (VRRs) for both UFs and AM [6–8].

While MWA is generally well-tolerated, some patients experience AEs that can affect their recovery and overall treatment experience. Adverse reactions such as pain, nausea and vomiting, vaginal discharge, and infection can significantly impact patients’ postoperative quality of life, while severe risks, including bleeding, adjacent organ injury (bowel, ureter), abnormal vital signs, and anesthesia-related complications directly threaten patients’ lives. However, existing research on the safety of MWA is limited, with most studies focusing on short-term outcomes or analyzing small patient cohorts [9, 10]. Furthermore, the risk factors contributing to AEs remain poorly understood, making it challenging to optimize procedural techniques and improve patient selection. “Do no harm” is the foremost principle of any medical intervention. Evaluating the safety of MWA technology is the basis for ensuring its overall benefits outweigh potential risks. Therefore, a more comprehensive evaluation of MWA-related AEs is crucial to refine clinical protocols, enhance patient safety, and expand the application of this method as a viable alternative to conventional surgical treatments.

This study aims to systematically analyze the incidence and characteristics of AEs following MWA for UFs and AM, identify the associated risk factors, and propose evidence-based recommendations. These objectives seek to refine technical operating protocols, optimize patient selection criteria, and develop preventive measures, thereby translating risk data into clinical decision-making tools, ultimately achieving effective symptom relief while controlling and minimizing risks to advance the safe application and widespread adoption of MWA.

## Materials and methods

This study was approved by the Ethics Committee of the Aerospace Center Hospital (2024-14-UD-AI). No data were obtained from publicly available databases.

### Research participants

**Fig. 1:**
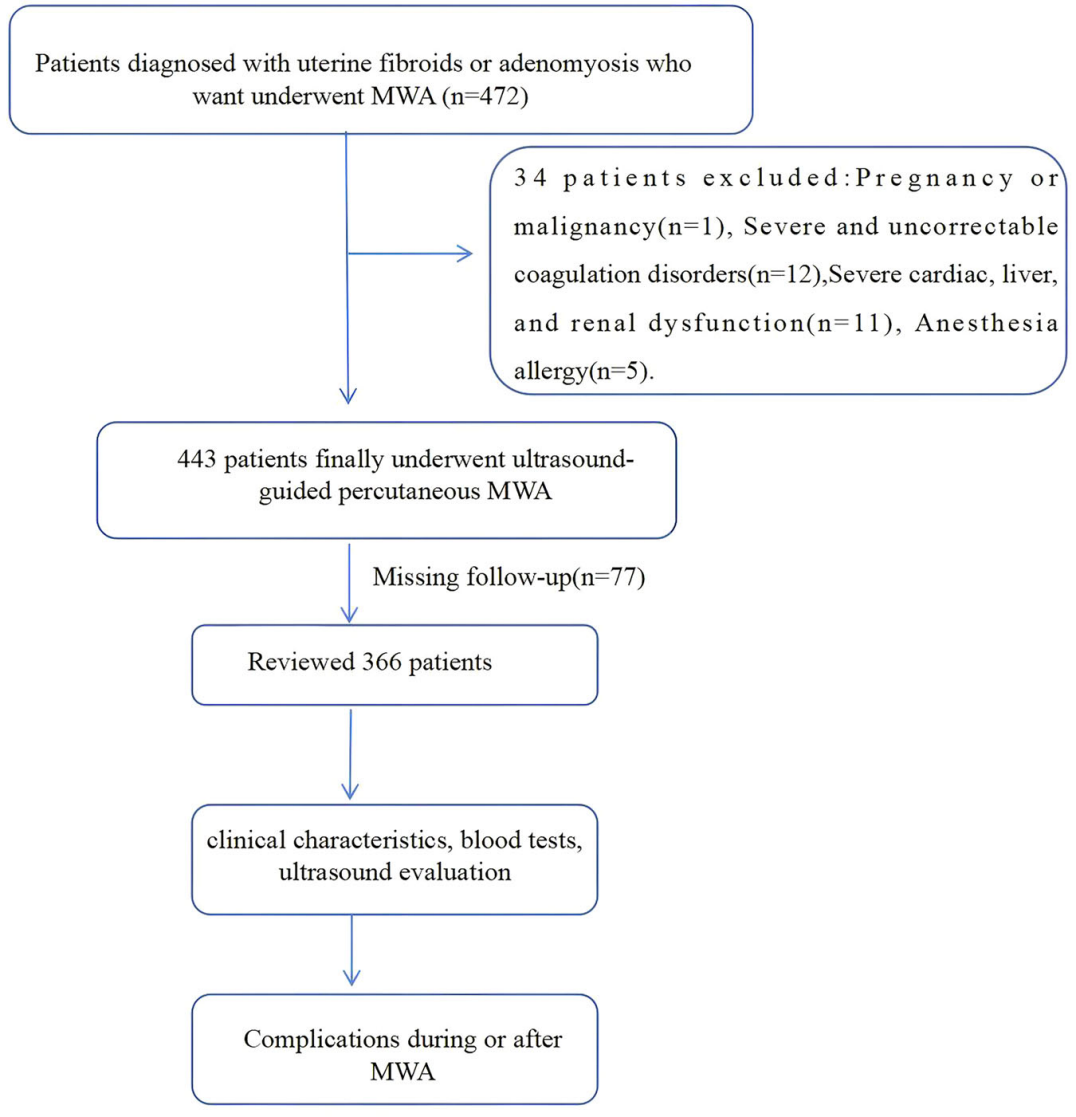
Flowchart of the whole workflow and recruitment of patients. MWA, microwave ablation

A total of 443 patients diagnosed with UFs or AM who underwent ultrasound-guided percutaneous microwave ablation (UPMWA) at the Aerospace Center Hospital, YiXian Hospital, and Emergency General Hospital between October 2023 and August 2024 were retrospectively reviewed. All patients voluntarily signed the informed consent form for interventional therapy with MWA. Figure 1 presents the flow chart of this study. All hospitals participating in this study underwent uniform training, strictly followed standardized MWA protocols, implemented consistent operational parameters, and adhered to a unified AEs reporting mechanism, ensuring the consistency of the research data.

The inclusion criteria were as follows:

- Confirmed diagnosis of UFs or AM based on pathology and ultrasound imaging;
- Presence of clinical symptoms, including abnormal uterine bleeding, secondary anemia, progressive dysmenorrhea, or uterine enlargement;
- Voluntary consent to undergo MWA;
- Availability of a safe and accessible transabdominal puncture pathway.

The exclusion criteria were as follows:

- Pregnancy or malignancy;
- Severe and uncorrectable coagulation disorders;
- Severe cardiac, liver, and renal dysfunction;
- Anesthesia allergy.

### Microwave ablation procedure

#### Instruments

The MWA procedure was performed using a Samsung ultrasound diagnostic system equipped with a convex array probe (SC5-1U). The ablation system consisted of an MAS-100A1 machine (Jiangsu u-nanocure Technology Co., Ltd) with a disposable MWA needle MAS-200A6 (size, F2.0 £ 17.0 mm) and an ECO-100A1 machine (YIGAO Microwave System Engineering Co., Ltd.) equipped with a disposable MWA needle ECO-100C18 (size, F2.0 £ 15 mm). The ultrasound contrast agents were SonoVue and Sonazoid.

#### Anesthesia procedures

Under local anesthesia, patients remain conscious and can provide feedback on any abnormal pain, allowing operators to adjust the dosage of anesthetics in real-time. Compared to general anesthesia, local anesthesia significantly reduces risks of complications such as respiratory depression [11]. Additionally, it offers advantages like low cost and rapid postoperative recovery, thus making it a preferred option for surgery [12]. All patients underwent local anesthesia in an inpatient setting. A mixture of 2% lidocaine (5 mL × 2 vials) diluted in saline to a total volume of 40 mL was percutaneously injected into the subserosal layer under ultrasound guidance for subserosal anesthesia.

#### Treatment procedure

**Fig. 2:**
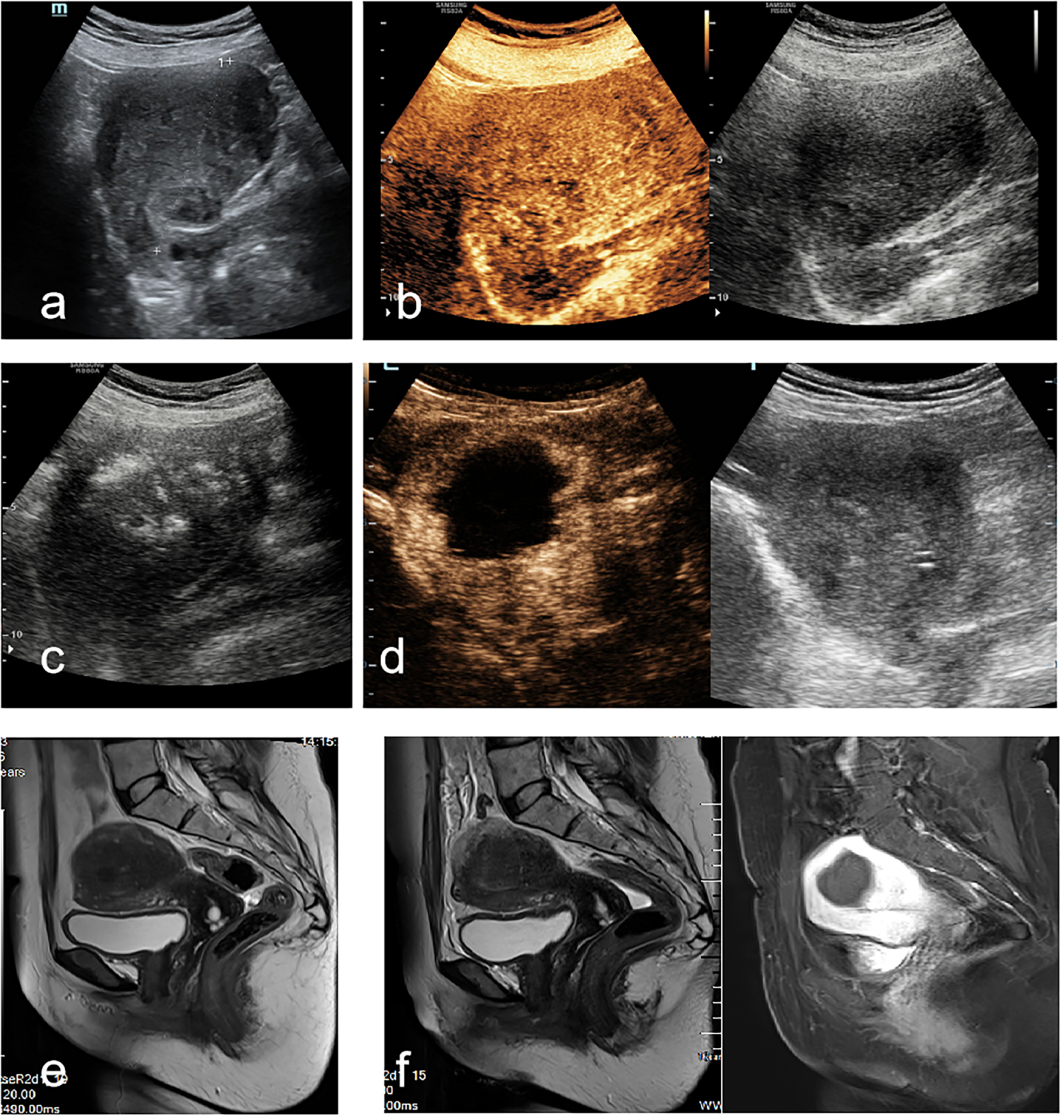
A 45-year-old woman with adenomyosis. **a** Preoperative two-dimensional ultrasound image showed the uterus size was 8.3 × 7.7 × 6.3 cm, volume 195.1 mL. **b** CEUS before ablation: contrast agent perfused in the entire uterus. **c** The microwave energy radiated from the emission tip. **d** Postoperative review on the day after MWA, CEUS was performed to assess the volume of the NPV. **e** MRI (‘T2WI): enlargement of the uterus. **f** After MWA, contrast-enhanced imaging demonstrated persistent non-enhancement within the ablation zone. CEUS, contrast-enhanced ultrasound; MWA, microwave ablation; NPV, the non-perfused volume

**Fig. 3:**
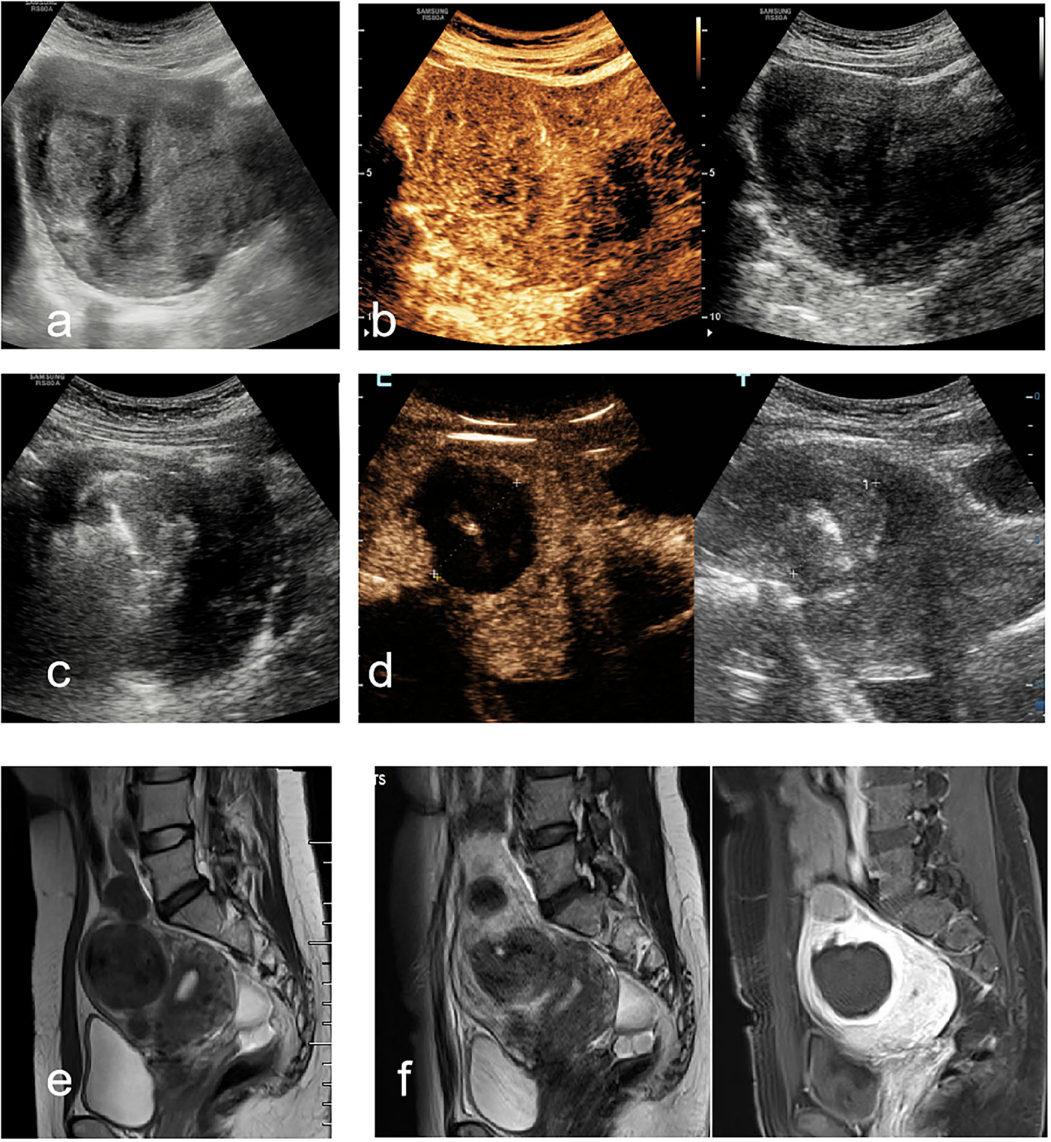
A 43-year-old woman with UFs. **a** Preoperative two-dimensional ultrasound image of UFs (FIGO V): 4.6 × 5.4 × 5.4 cm, volume 68.6 mL. **b** CEUS before ablation: initial perfusion of the pseudo-capsule, followed by enhancement of the entire lesion. **c** The microwave energy radiated from the emission tip. **d** Postoperative review on the day after MWA using CEUS: complete ablation achieved (ablation rate 80%). **e** MRI (‘T2WI): the FIGO V in the anterior wall. **f** After MWA, contrast-enhanced MRI demonstrated persistent non-enhancement within the ablation zone. UFs, uterine fibroids; CEUS, contrast-enhanced ultrasound; MWA, microwave ablation; FIGO, the International Federation of Gynecology and Obstetrics classification

A safe and minimally invasive transabdominal puncture pathway was carefully selected to avoid injury to adjacent organs such as the bladder and rectum. To enhance safety and visualization, artificial ascites was created prior to ablation [13]. The microwave therapy device was activated, and the ablation needle was percutaneously inserted into the uterine myometrial lesion. The power settings ranged from 40 to 60 W, and both fixed-point ablation and moving multi-point ablation techniques were used depending on lesion characteristics. Real-time monitoring was performed to track the formation of vaporized hyperechoic signals at the lesion site, and ablation was terminated when the hyperechoic zone reached 0.3–0.5 cm from the lesion margins. Under ultrasound contrast guidance, check for any bleeding or fluid accumulation in the pelvis and provide supplementary treatment for areas where ablation is insufficient. Postoperative magnetic resonance imaging (MRI) was performed to confirm the ablation effect (Figs. 2 and  3).

### Assessment indicators

All patients completed a preoperative symptom severity questionnaire, and the transformed symptom severity score (tSSS) was calculated using the formula: tSSS = (actual original score − 8)/32 × 100 [14]. Preoperative imaging evaluations included ultrasonography and contrast-enhanced ultrasonography (CEUS) to determine lesion size, number, location, and type. The lesion volume (V) was calculated as follows: *V* = 0.523 × D1 × D2 × D3 (D1, the maximum transverse diameter of the fibroid; D2 and D3, 2 diameters perpendicular to D1). Pathological confirmation was performed for lesion classification, and fibroids were categorized based on the International Federation of Gynecology and Obstetrics (FIGO) Classification [15].

Routine laboratory tests were conducted one week before and after MWA to evaluate hematological and organ function parameters, including red blood cell count (RBC), hemoglobin (HGB), albumin (ALB), total bilirubin (TBIL), direct bilirubin (DBIL), indirect bilirubin (IDBIL), aspartate aminotransferase (AST), alanine aminotransferase (ALT), and creatinine (CREA).

During the procedure, the patient’s vital signs (blood pressure, heart rate, etc.), pain at the surgical site, total ablation time, number of ablation lesions, and ablation range were recorded. Pain during MWA was assessed by trained assistants using the numeric rating scale (NRS). Patients were stratified by peak intraoperative pain scores: Mild: 1–3 points, Moderate-to-Severe: ≥ 4 points [16]. Immediately after the treatment, CEUS was performed to confirm the ablation range by assessing non-perfused target areas, and the non-perfused volume (NPV) was calculated. The ablation time for per unit volume (TPV) was determined using the formula: TPV = Ablation time/NPV.

### Patient tolerance evaluation

Postoperative AEs were systematically recorded, including abdominal pain, vaginal discharge, changes in blood pressure or heart rate during UPMWA, fever, nausea or vomiting, hematuria, and intestinal injury. All centers consistently used Loxoprofen for some patients with unbearable abdominal pain after MWA; minor variations were limited to individual dose adjustments based on patient-specific conditions. Persistent symptoms were continuously monitored until they resolved.

### Statistical analysis

Statistical analyses were performed using SPSS 22.0 software. Quantitative variables were described using mean ± standard deviation, and non-normally distributed data were presented as median and interquartile range. Qualitative data were described as frequencies and percentages. Comparisons between groups were conducted using a *t*-test or Chi-Square test. Logistic regression analysis was utilized to identify the main factors associated with adverse events. A *p* < 0.05 was considered statistically significant.

## Results

### Clinical characteristics

A total of 443 patients underwent UPMWA, and 366 patients were included in this study. Additionally, 12 patients refused to participate in the postoperative AEs questionnaire survey despite multiple follow-up attempts. A further 35 patients were excluded for lacking critical data: 14 lacked preoperative tSSS assessments, 12 had incomplete intraoperative operation information, and 10 did not undergo pathological biopsy confirmation. Table 1 presents a detailed comparison of baseline characteristics between the 77 patients lost to follow-up and the remaining 366 included patients. The detailed process of patient selection is presented in Fig. 1. Among these patients, 301 were diagnosed with UFs and 65 with AM. The mean age of the patients was 43.76 ± 5.50 years, with no significant difference between the UFs and AM groups (*p* = 0.367). Compared to UFs patients, those with AM had significantly lower TPV (3.63 ± 2.65 vs. 6.65 ± 4.00 s/mL, *p* < 0.001), shorter ablation time (575.86 ± 367.55 vs. 869.57 ± 497.56 s, *p* < 0.001) and higher tSSS (47.25 ± 17.68 vs. 31.06 ± 18.66, *p* < 0.001). Moderate-to-severe pain during MWA was more common in AM patients (66.15% vs. 20.60%, *p* < 0.001), and they were more likely to have single ablation lesions (80.00% vs. 44.85%, *p* < 0.001). Additionally, AM patients had significantly lower NPV values compared to those with UFs (90.03 ± 77.44 vs. 125.87 ± 112.19, *p* = 0.027). According to the FIGO classification, 87.58% of UFs cases were classified as FIGO 4–6, while FIGO 0–3 accounted for 12.42%. A comprehensive summary of patient demographics, preoperative characteristics, and intraoperative details is provided in Table 1.

**Table 1:** Characteristics of the patients

Variables (mean ± SD)	No follow-up (*n* = 77)	Total (*n* = 366)	UFs (*n* = 301)	AM (*n* = 65)	Statistic	*p*
Age (year)	43.57 ± 5.31	43.76 ± 5.50	43.88 ± 5.54	43.20 ± 5.34	*t* = 0.90	0.367
TPV (s/mL)	5.97 ± 3.68	6.11 ± 3.97	6.65 ± 4.00	3.63 ± 2.65	*t* = 7.52	**< 0.001**
Ablation time (s)	823.17 ± 479.29	817.41 ± 489.69	869.57 ± 497.56	575.86 ± 367.55	*t* = 4.50	**< 0.001**
Volume (mL)	279.28 ± 835.14	275.16 ± 931.54	286.88 ± 1022.67	220.86 ± 207.27	*t* = 0.52	0.605
tSSS	-	33.97 ± 19.48	31.06 ± 18.66	47.25 ± 17.68	*t* = -6.04	**< 0.001**
NPV (mL)	120.17 ± 108.32	119.08 ± 107.28	125.87 ± 112.19	90.03 ± 77.44	*t* = 2.23	**0.027**
Pain during MWA, *n* (%)					*χ*² = 54.23	**< 0.001**
Moderate-to-Severe	-	105 (28.69)	62 (20.60)	43 (66.15)		
Mild	-	261 (71.31)	239 (79.40)	22 (33.85)		
Ablation lesions *n* (%)					*χ*² = 26.43	**< 0.001**
Single	38 (49.35)	187 (51.09)	135 (44.85)	52 (80.00)		
Multiple	39 (50.65)	179 (48.91)	166 (55.15)	13 (20.00)		
FIGO classification, *n* (%)						
FIGO 4–6	53 (68.83)	264 (72.13)	264 (87.70)	/		
FIGO 0–3	9 (11.69)	37 (10.11)	37 (12.30)	/		
AM	15 (19.48)	65 (17.76)	/	65 (100.00)		
Disease type, *n* (%)						
UFs	62 (80.52)	301 (82.24)	301 (100.00)	0 (0.00)		
AM	15 (19.48)	65 (17.76)	0 (0.00)	65 (100.00)		

Continuous quantitative variables were described using mean ± standard deviation (SD), and other data were described as frequencies and percentages

*UFs* uterine fibroids, *AM* adenomyosis, *TPV* the ablation time for per unit volume, *tSSS* transformed symptom severity score, *NPV* the non-perfused volume, *FIGO* the International Federation of Gynecology and Obstetrics classification

The statistically significant values (*p* < 0.05) were represented in bold font

### Evaluation of adverse events

**Fig. 4:**
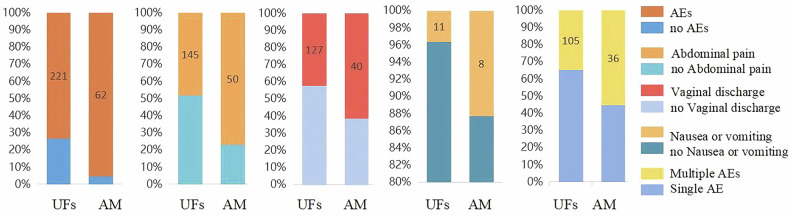
Compared with the UFs patients, the AM patients had a higher overall AEs rate, including abdominal pain, vaginal discharge, and Nausea or vomiting. The probability of multiple complications occurring simultaneously in the AM patients was significantly higher than that in the UFs patients. UFs, uterine fibroids; AM, adenomyosis; AEs, adverse events

The overall AEs rate of benign uterine diseases was 77.32% (283/366). Patients with AM exhibited a significantly higher AE rate compared to those with UFs (95.38% vs. 73.42%, *p* < 0.001). Specifically, AM patients experienced higher rates of abdominal pain (76.92% vs. 4.8.17%, *p* < 0.001), vaginal discharge (61.54% vs. 42.19%, *p* = 0.005), and Nausea or vomiting (12.31% vs. 3.65%, *p* = 0.011) (Table 2 and Fig. 4). However, no significant difference was observed AM and UFs patients in other AEs such as changes in blood pressure or heart rate, fever, and hematuria. Additionally, multiple concurrent AEs were significantly more frequent in AM patients than in UFs patients (55.38% vs. 34.88%, *p* = 0.002). The co-occurrence rate of AEs involving abdominal pain combined with vaginal discharge was significantly higher than that of others, both in AM (80%, 29/36) and UFs (66.67%, 70/105). All AEs were classified as grade 1 or 2 according to the Cirse Classification System (CIRSE), and no patients experienced severe or permanent AEs.

**Table 2:** Occurrence of AEs for UFs and AM following MWA

Variables/*n* (%)	Total (*n* = 366)	UFs (*n* = 301)	AM (*n* = 65)	Statistic	*p*
AEs, *n* (%)	283 (77.32)	221 (73.42)	62 (95.38)	*χ*² = 14.71	**< 0.001**
Multiple AEs, *n* (%)	141 (38.52)	105 (34.88)	36 (55.38)	*χ*² = 9.49	**0.002**
Abdominal pain, *n* (%)	195 (53.28)	145 (48.17)	50 (76.92)	*χ*² = 17.75	**< 0.001**
Vaginal discharge, *n* (%)	167 (45.63)	127 (42.19)	40 (61.54)	*χ*² = 8.06	**0.005**
Changes of blood pressure or heart rate, *n* (%)	71 (19.40)	55 (18.27)	16 (24.62)	*χ*²= 1.38	0.241
Nausea or vomiting, *n* (%)	19 (5.19)	11 (3.65)	8 (12.31)	*χ*² = 8.13	**0.004**
Fever, *n* (%)	12 (3.28)	11 (3.65)	1 (1.54)	*χ*² = 0.75	0.385
Hematuria, *n* (%)	2 (0.55)	1 (0.33)	1 (1.54)	*χ*² = 1.43	0.232
CIRSE Grade 1	349 (95.36)	289 (96.00)	60 (92.31)	*χ*² = 1.657	0.1980
CIRSE Grade 2	17 (4.64)	12 (4.00)	5 (7.69)	*χ*² = 1.657	0.1980
CIRSE Grade 3–5	0	0	0	-	-

Data was described as frequencies and percentages

Statistical tests such as chi-square analysis could not be performed when zero frequencies were present

*UFs* uterine fibroids, *AM* adenomyosis, *AEs* adverse events, *MWA* microwave ablation

The statistically significant values (*p* < 0.05) were represented in bold font

### Factors associated with adverse events

All cases were divided into two groups based on the presence or absence of AEs (Table 2): no AEs (83 cases) and AEs (283 cases). Univariate logistic regression analysis showed that tSSS, pain during MWA, FIGO classification, and disease type were associated with the occurrence of AEs. Multivariate logistic regression analyses indicated AM (OR = 3.77, *p* = 0.039) and higher tSSS scores (tSSS 25–40: OR = 2.98, *p* = 0.007, tSSS > 40: OR = 2.36, *p* = 0.022) were independent risk factors for AEs. Age (*p* = 0.213), TPV (*p* = 0.262), ablation time (*p* = 0.794), volume (*p* = 0.901), NPV (*p* = 0.750) and ablation lesions (*p* = 0.111) were not significantly associated with AEs (Table 3).

**Table 3:** Univariate and multivariate analysis for the risk factors of AEs following MWA

Vriables			Univariate analysis		Multivariate analysis	
	No AEs (*n* = 83)	AEs (*n* = 283)	*p*	OR (95% CI)	*p*	OR (95% CI)
Age (year)	45.00 (42.00, 47.00)	44.00 (41.00, 47.00)	0.213	0.73 (0.44–1.20)		
TPV (s/mL)	5.67 (3.89, 7.96)	5.37 (3.32, 7.60)	0.262	0.75 (0.46–1.23)		
Ablation time (s)	728.00 (492.50, 1065.00)	720.00 (458.00, 1074.00)	0.794	0.94 (0.57–1.53)		
Volume (mL)	162.75 (106.20, 258.10)	162.48 (89.18, 259.77)	0.901	0.97 (0.59–1.58)		
NPV (mL)	79.20 (53.82, 152.77)	84.20 (45.90, 160.10)	0.750	1.10 (0.62–1.92)		
tSSS						
0–18	26 (35.62)	39 (15.60)				
18–25	16 (21.92)	54 (21.60)	**0.033**	2.25 (1.07–4.75)	0.057	2.09 (0.98–4.46)
25–40	13 (17.81)	63 (25.20)	**0.003**	3.23 (1.49–7.02)	**0.007**	2.98 (1.35–6.57)
> 40	18 (24.66)	94 (37.60)	**< 0.001**	3.48 (1.72–7.06)	**0.022**	2.36 (1.13–4.95)
Pain during MWA						
Mild	71 (85.54)	190 (67.14)				
Moderate-to-severe	12 (14.46)	93 (32.86)	**0.002**	2.90 (1.50–5.60)	0.058	2.16 (0.97–4.81)
Ablation lesions						
Single	36 (43.37)	151 (53.36)				
Multiple	47 (56.63)	132 (46.64)	0.111	0.67 (0.41–1.10)		
FIGO classification						
FIGO 4–6	74 (89.16)	190 (67.14)				
FIGO 0–3	2 (2.41)	35 (12.37)	**0.041**	2.76 (1.04–7.32)		
AM	7 (8.43)	58 (20.49)	**< 0.001**	7.63 (2.32–25.11)		
Disease type						
UFs	80 (96.39)	221 (78.09)				
AM	3 (3.61)	62 (21.91)	**< 0.001**	7.48 (2.28–24.50)	**0.039**	3.77 (1.07–13.32)

Non-normative distributed data, represented as median (25–75% interquartile range), and other data are represented as numbers (percentages)

*AEs* adverse events, *UFs* uterine fibroids, *AM* adenomyosis, *TPV*, the ablation time for per unit volume, *tSSS* transformed symptom severity score, *NPV* the non-perfused volume, *FIGO* the International Federation of Gynecology and Obstetrics classification, *OR* odds ratio, *CI* confidence Interval

The statistically significant values (*p* < 0.05) were represented in bold font

### Factors associated with AEs in UFs

In the subgroup analysis of UFs patients, both higher tSSS (tSSS 25–40: OR = 2.85, *p* = 0.01, and tSSS >  40: OR = 2.6, *p* = 0.014) and moderate-to-severe pain during MWA (OR = 2.35, *p* = 0.048) were significantly associated with the occurrence of AEs (Table 4).

**Table 4:** Factors related to AEs of UFs

Variables			Univariate analysis		Multivariate analysis	
	*no AEs (n* = *80)*	*AEs (n* = *221)*	*p*	OR (95% CI)	*p*	OR (95% CI)
Age (year)	45.00 (41.75, 47.00)	44.00 (41.00, 48.00)	0.473	0.83 (0.49–1.39)		
Ablation time (s)	724.00 (493.75, 1077.00)	758.00 (531.00, 1123.00)	0.578	1.16 (0.69–1.93)		
Volume (mL)	162.36 (106.89, 255.35)	160.05 (92.81, 260.03)	0.821	0.94 (0.57–1.57)		
TPV (s/mL)	5.73 (4.07, 8.08)	5.84 (4.22, 8.16)	0.972	1.01 (0.61–1.68)		
NPV (mL)	78.60 (53.85, 149.85)	96.20 (52.32, 174.85)	0.275	1.39 (0.77–2.52)		
tSSS						
0–18	26 (37.14)	38 (19.49)				
18–25	16 (22.86)	46 (23.59)	0.080	1.97 (0.92–4.19)	0.069	2.03 (0.95–4.36)
25–40	13 (18.57)	52 (26.67)	**0.012**	2.74 (1.25–6.01)	**0.010**	2.85 (1.29–6.30)
> 40	15 (21.43)	59 (30.26)	**0.010**	2.69 (1.26–5.73)	**0.014**	2.60 (1.21–5.57)
Pain during MWA						
Mild	70 (87.50)	169 (76.47)				
Moderate-to-severe	10 (12.50)	52 (23.53)	**0.040**	2.15 (1.04–4.48)	0.054	2.35 (0.98–5.61)
Ablation lesions						
Single	34 (42.50)	101 (45.70)				
Multiple	46 (57.50)	120 (54.30)	0.622	0.88 (0.52–1.47)		
FIGO classification						
FIGO 4–6	75 (93.75)	189 (85.52)				
FIGO 0–3	5 (6.25)	32 (14.48)	0.062	2.54 (0.95–6.77)		

Non-normative distributed data, represented as median (25–75% interquartile range), and other data are represented as numbers (percentages)

*UFs* uterine fibroids, *TPV* the ablation time for per unit volume, *tSSS* transformed symptom severity score, *NPV* the non-perfused volume, *FIGO* the International Federation of Gynecology and Obstetrics classification, *OR* odds ratio, *CI* confidence interval

The statistically significant values (*p* < 0.05) were represented in bold font

### Risk factors for common AEs in UFs

To further analyze specific AEs, patients with UFs were divided into two groups based on the presence or absence of three common AEs: abdominal pain (48.17%), vaginal discharge (42.19%), and changes in blood pressure or heart rate (18.27%). For postoperative abdominal pain, moderate to severe pain during MWA (OR = 3.63, *p* < 0.001) and higher tSSS scores (25–40: OR = 2.12, *p* = 0.043) were identified as significant risk factors (Table 5). In terms of vaginal discharge, although multivariate regression analysis showed that only higher tSSS scores (tSSS 25–40: OR = 3.22, *p* = 0.003, tSSS > 40: OR = 3.32, *p* = 0.001) were independent risk factors, univariate analysis suggested that vaginal discharge risk also increased with FIGO 0–3 (OR = 2.53, *p* = 0.010). (Supplementary Table S1). For changes in blood pressure or heart rate, univariate logistic regression analysis showed that moderate-to-severe pain during MWA was associated with the changes of blood pressure or heart rate(moderate to severe: OR = 2.00, *p* = 0.039, Supplementary Table S2).

**Table 5:** Risk factors for Abdominal pain in UFs

Variables			Univariate analysis		Multivariate analysis	
	no Abdominal pain (*n* = 156)	Abdominal pain (*n* = 145)	*p*	OR (95% CI)	*p*	OR (95% CI)
Age (year)	45.00 (42.00, 48.00)	44.00 (41.00, 47.00)	0.093	0.68 (0.43–1.07)		
Ablation time (s)	740.00 (493.75, 1090.00)	758.00 (544.00, 1118.00)	0.602	1.13 (0.72–1.77)		
Volume (mL)	151.09 (95.17, 255.35)	165.88 (94.46, 260.03)	0.452	1.19 (0.76–1.87)		
TPV (s/mL)	6.29 (4.86, 8.84)	5.54 (3.77, 7.68)	0.075	0.66 (0.42–1.04)		
NPV (mL) median	85.20 (52.93, 158.58)	96.60 (53.90, 171.40)	0.552	1.17 (0.70–1.96)		
tSSS						
0–18	41 (29.50)	23 (18.25)				
18–25	33 (23.74)	29 (23.02)	0.218	1.57 (0.77–3.20)	0.174	1.67 (0.80–3.47)
25–40	31 (22.30)	34 (26.98)	0.062	1.96 (0.97–3.96)	**0.043**	2.12 (1.02–4.38)
> 40	34 (24.46)	40 (31.75)	**0.034**	2.10 (1.06–4.16)	**0.054**	2.00 (0.99–4.06)
Pain during MWA						
Mild	137 (87.82)	102 (70.34)				
Moderate-to-Severe	19 (12.18)	43 (29.66)	**< 0.001**	3.04 (1.67–5.53)	**< 0.001**	3.63 (1.80–7.35)
Ablation lesions						
Single	70 (44.87)	65 (44.83)				
Multiple	86 (55.13)	80 (55.17)	0.994	1.00 (0.64–1.58)		
FIGO classification						
FIGO 4–6	138 (88.46)	126 (86.90)				
FIGO 0–3	18 (11.54)	19 (13.10)	0.680	1.16 (0.58–2.30)		

Non-normative distributed data, represented as median (25–75% interquartile range), and other data are represented as numbers (percentages)

*UFs* uterine fibroids, *TPV* the ablation time for per unit volume, *tSSS* transformed symptom severity score, *NPV* the non-perfused volume, *FIGO* the International Federation of Gynecology and Obstetrics classification, *OR* odds ratio, *CI* confidence interval

The statistically significant values (*p* < 0.05) were represented in bold font

### Comparison of peripheral blood test indexes before and after MWA

Postoperative blood test results showed significant increases in AST (19.26 ± 0.4227 to 24.77 ± 0.5768 IU/L, *p* < 0.0001), AST/ALT (1.306 ± 0.0283 to 1.979 ± 0.0763, *p* < 0.0001), CREA (55.99 ± 0.5917 to 57.51 ± 0.8345 umol/L, *p* = *0.0290*), TBIL (10.57 ± 0.3329 to 13.88 ± 0.4715 umol/L, *p* < 0.0001), DBIL (1.785 ± 0.0559 to 2.320 ± 0.0757 umol/L, *p* < 0.0001) and IDBIL (8.780 ± 0.2821 to 11.56 ± 0.4022 umol/L, *p* < 0.0001). Meanwhile, a significant decrease in the levels of ALB (42.03 ± 0.1619 to 37.49 ± 0.1678 g/L, *p* < 0.0001), HGB (111.0 ± 1.607 to 106.3 ± 1.56 g/L, *p* < 0.0001) and RBC (4.083 ± 0.0296 to 3.935 ± 0.0292 ×  10^12^/L, *p* < 0.0001) was observed. Among these, only a small proportion of patients exhibited clinically abnormal elevations in liver and kidney function markers: AST (10.00%), AST/ALT ratio (6.19%), CREA (0.476%), TBIL (15.24%), and IDBIL (14.76%). Additionally, reductions of more than 10% in ALB, RBC, and HGB levels were observed in 42.38%, 13.33%, and 15.24% of patients, respectively (Supplementary Table S3).

## Discussion

MWA has demonstrated significant advantages in the treatment of UFs and AM because of its minimal invasiveness and precise targeting of lesions. This procedure effectively reduces lesion sizes and alleviates clinical symptoms, with a lower risk of AEs, such as infection and bleeding, than traditional surgical approaches [7, 8, 17–19]. However, despite these benefits, AEs still occur during the clinical management of benign uterine lesions. Currently, research on MWA-related AEs and their risk factors remains limited and is often based on small-scale preliminary studies [9, 10].

The common AEs after both MWA, HIIFU and RFA treatments were abdominal pain (53.28% vs. 62.3% and 40.0%), vaginal discharge (16.2% vs. 45.63% and 50.0%) [20, 21]. Compared to HIFU, MWA demonstrates a lower incidence of abdominal pain and skin burns, and none of the patients experienced severe or permanent AEs [21]. Consistent with previous studies, the incidence rates of abdominal pain (76.92% vs. 71.0% and 55.6%) and vaginal discharge (61.54% vs. 58.9% and 47.2%) were relatively high in patients with AM, whereas the incidence rates of other adverse reactions, such as fever (1.54% vs. 9.3% and 41.7%) and nausea and/or vomiting (12.31% vs. 19.4%), were low [17, 18]. However, prior studies were often limited to small sample sizes or focused on AM in the posterior uterine wall, omitting certain AEs such as changes in blood pressure or heart rate and hematuria.

Among the risk factors analyzed, AM and higher tSSS were significantly associated with AEs. The increased AE rate in patients with AM compared with that in patients with UFs may be attributed to the unique pathological characteristics of AM. MWA induces irreversible coagulative necrosis and microvascular damage in ablation lesions through a local thermal effect, which can lead to the contraction of uterine muscle fibers [22]. AM lesions are typically extensive, characterized by the invasion and proliferation of endometrial tissue within the uterine muscle layer, and are usually accompanied by increased microvessels and local inflammatory responses [2, 23, 24]. These factors can lead to greater procedural discomfort, increased sensitivity to thermal injury, and a higher likelihood of nausea or vomiting. Additionally, owing to the absence of a clear boundary between AM and normal tissue, controlling the extent of ablation is more challenging, increasing the risk of endometrial injury and subsequent vaginal discharge. The occurrence of vaginal discharge in AM is significantly related to the minimum distance from the endomyometrial junction margin to the non-perfused lesion [25]. Therefore, preoperative safety counseling and intraoperative modifications are critical for optimizing the treatment of patients with AM undergoing MWA.

Similar to AM and previous findings, abdominal pain and vaginal discharge were the most frequent AEs in patients with UF [7, 26, 27], along with changes in blood pressure or heart rate. Our analysis also identified higher tSSS as a significant risk factor for multiple AEs, suggesting that some postoperative symptoms may represent a continuation of preoperative clinical manifestations rather than new AEs and may gradually resolve as treatment efficacy improves. We found that the OR for the highest tSSS (> 40) is lower than for the middle category (25–40) when both are compared to lower tSSS. The relationship between tSSS and AEs may not follow a strictly linear pattern. Patients with long-standing severe symptoms (tSSS > 40) may develop heightened tolerance to AEs, potentially leading to under-reporting of mild AEs. We also consider whether patients with tSSS > 40 might have received pre-procedural interventions (e.g., pain medications, anti-inflammatory drugs) to alleviate severe symptoms, which could modestly reduce their procedural AE risk. Importantly, we emphasize that despite this minor difference in OR, higher tSSS (25–40 and > 40) remain statistically significant independent risk factors for AEs. Further research is needed to confirm this hypothesis and explore whether preoperative symptom severity can serve as a predictive marker for post-treatment recovery trajectories, ultimately aiding in individualized patient counseling and optimizing post-procedural management.

Additionally, in the present study, moderate-to-severe pain during MWA was strongly associated with overall AEs, postoperative abdominal pain, and changes in blood pressure or heart rate, which may be linked to the different anesthesia methods used during MWA procedures [28, 29]. Currently, the clinical choice of the anesthesia method is primarily determined by patient tolerance and operator preference, with local anesthesia being the most common approach. While some studies have suggested that general anesthesia in MWA for hepatocellular carcinoma increases costs and AE rates [30], others reported no significant differences in AEs across anesthesia methods in endometrial MWA [31]. However, research on anesthesia-related AEs in patients with UFs undergoing MWA is lacking. Our findings underscore the need for further investigation of anesthesia optimization to improve patient comfort and reduce procedural AEs.

**Fig. 5:**
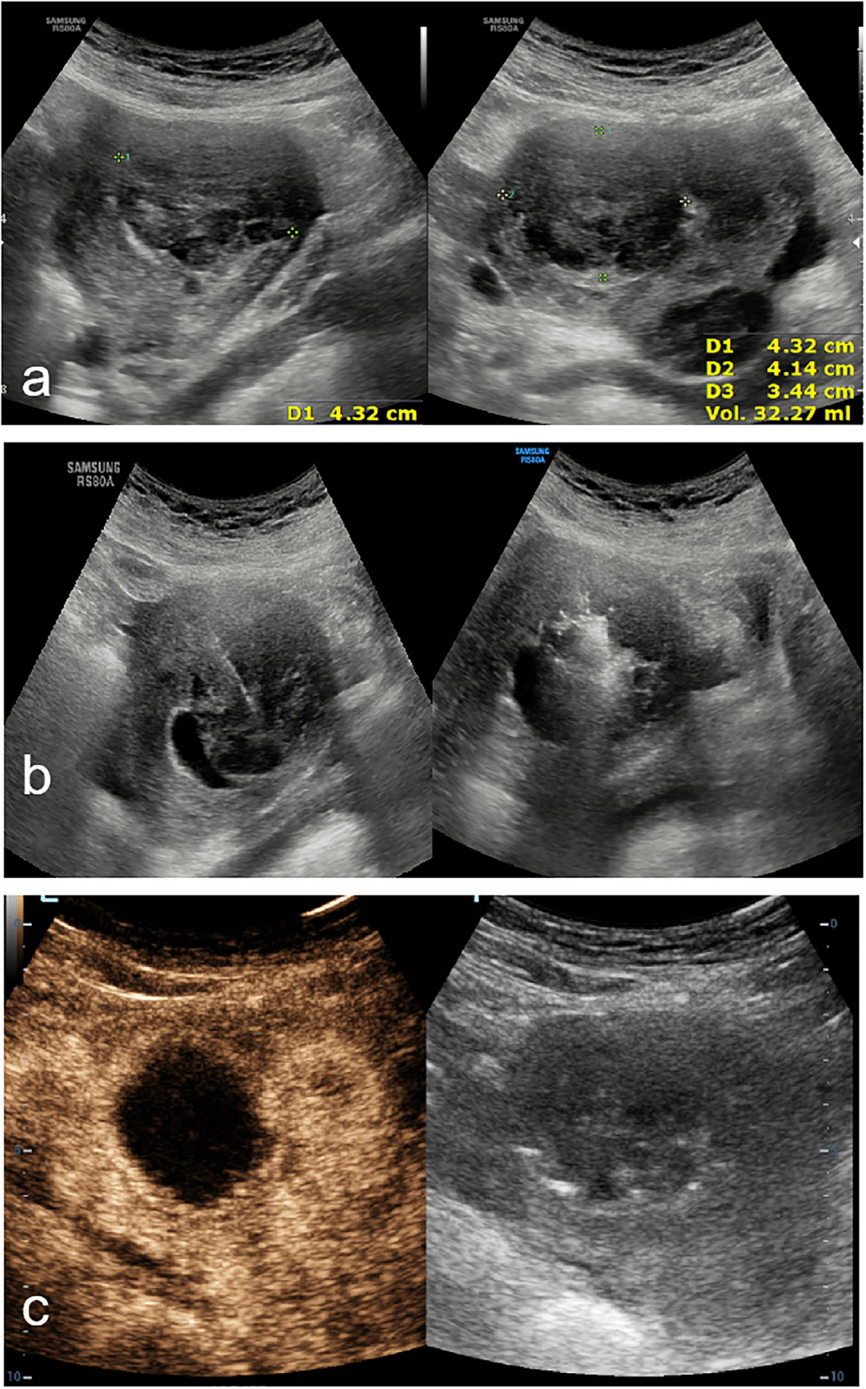
A case of submucosal myoma was treated with transcutaneous intrauterine infusion of chilled saline instillation, and no vaginal discharge occurred after the MWA. **a** Preoperative images of UFs (FIGO II): 4.3 × 4.1 × 3.4 cm, volume 32.27 mL. **b** During MWA, an 18-gauge puncture needle was percutaneously penetrated into the uterine cavity under the guidance of ultrasound, and the outer end of the needle was supplied with chilled saline for instillation, the instilled normal saline was discharged through the vagina. **c** Postoperative review on the day after MWA. MWA, microwave ablation; FIGO the International Federation of Gynecology and Obstetrics classification

The results of the present study identified a potential association between FIGO types 0–3 and the occurrence of vaginal discharge, although this relationship was significant only in the univariate analysis and was not confirmed in the multivariate analysis. This suggests that while FIGO 0–3 UFs may be linked to a higher likelihood of post-procedural discharge, other factors, such as tSSS and intraoperative conditions, may play more decisive roles. This trend may be attributed to the proximity of FIGO 0–3 fibroids to the endometrium, which could increase the risk of thermal injury during ablation, resulting in vaginal discharge after the procedure [32–34]. Evidence suggests FIGO type 1-3 uterine fibroids confer elevated risks of graded endometrial injury, with 65.4%, 23.1%, and 11.5% patients exhibiting grades 0, 1, and 2 endometrial impairment, respectively [30]. Clinical approaches to prevent endometrial injury include increasing the distance between the ablation margin and endometrium and infusing cold saline into the uterine cavity to reduce the thermal effects [35, 36]. At our center, we use a puncture needle for transcutaneous intrauterine infusion of chilled saline, which effectively reduces endometrial damage (Fig. 5). Further research is warranted to clarify the specific role of FIGO classification in post-MWA AEs and determine the optimal protective measures for preserving endometrial integrity.

The observed perioperative changes in blood biomarkers were largely consistent with those reported previously [37]. Although some patients exhibited temporary abnormalities in the levels of markers associated with liver and kidney function, the proportions remained low and did not require clinical intervention, further supporting the safety profile of MWA for the treatment of benign uterine diseases.

This study has several limitations. First, the retrospective design may have introduced a selection bias, and the confounding factors include inter-operator variability and potential under-reporting of minor AEs. Second, AEs such as intestinal injury and superficial burns were not observed in our cohort, limiting our ability to analyze these rare but clinically significant AEs. Additionally, owing to the absence of long-term follow-up, the data on postoperative pregnancy outcomes and ovarian function were incomplete, preventing an in-depth evaluation of the impact of MWA on reproductive health.

In conclusion, despite the high incidence of AEs, no severe or permanent complications were observed, reinforcing the safety profile of MWA in gynecological practice. Notably, moderate-to-severe pain during MWA was strongly associated with AE occurrence, highlighting the need for further optimization of procedural and anesthesia protocols. By distinguishing AE risks between UFs and AM, our findings offer valuable insights into individualized treatment strategies. Future research should explore anesthesia strategies, endometrial protection techniques, and long-term reproductive outcomes to further refine MWA treatment in gynecological practice.

## Supplementary information

ELECTRONIC SUPPLEMENTARY MATERIAL

## Data Availability

The data and materials contained in this article were available.

## Abbreviations

AEs: Adverse events
AM: Adenomyosis
CEUS: Contrast-enhanced ultrasound
FIGO: The International Federation of Gynecology and Obstetrics Classification
MWA: Microwave ablation
NPV: The non-perfused volume
TPV: The ablation time for per unit volume
tSSS: Transformed symptom severity score
UFs: Uterine fibroids
