# Supplementary material for: Safety of percutaneous microwave ablation under local anesthesia for uterine fibroids and adenomyosis
Source: Insights Imaging. 2025 Dec 2;16:266. doi: 10.1186/s13244-025-02149-5 (PMC12672976; doi:10.1186/s13244-025-02149-5)
Supplement: Supplementary file 1 — ELECTRONIC SUPPLEMENTARY MATERIAL [file 13244_2025_2149_MOESM1_ESM.pdf]

Safety of Percutaneous Microwave Ablation under Local Anaesthesia for Uterine Fibroids and Adenomyosis

ELECTRONIC SUPPLEMENTARY MATERIAL

Supplementary Table S1 Risk factors for Vaginal discharge in UFs

Variables	*no* Vaginal discharge (*n = 174*)	Vaginal discharge (*n = 127*)	Univariate analysis		Multivariate analysis	
			P	OR (95%CI)	*P*	OR (95%CI)
Age(year)	44.00 (41.00, 48.00)	45.00 (41.00, 47.50)	0.949	0.99 (0.62 ~ 1.56)		
Ablation time (s)	777.50 (526.25, 1100.00)	726.00 (521.00, 1138.00)	0.272	0.77 (0.49 ~ 1.22)		
Volume(ml)	171.52 (104.34, 277.48)	140.29 (76.43, 236.28)	0.118	0.69 (0.44 ~ 1.10)		
TPV(s/ml)	5.67 (3.93, 7.58)	6.21 (4.49, 8.62)	0.217	1.33 (0.84 ~ 2.11)		
NPV(ml)	96.20 (54.57, 170.75)	84.00 (46.10, 146.65)	0.322	0.77 (0.45 ~ 1.30)		
tSSS						
0 -18	48 (32.00)	16 (13.91)				
18-25	38 (25.33)	24 (20.87)	0.100	1.89 (0.88 ~ 4.06)	0.108	1.87 (0.87 ~ 4.01)
25-40	30 (20.00)	35 (30.43)	**0.001**	3.50 (1.66 ~ 7.39)	**0.003**	3.22 (1.50 ~ 6.91)
>40	34 (22.67)	40 (34.78)	**<.001**	3.53 (1.71 ~ 7.30)	**0.001**	3.32 (1.59 ~ 6.94)
Pain during MWA						
Mild	142 (81.61)	97 (76.38)				
Moderate-to-Severe	32 (18.39)	30 (23.62)	0.269	1.37 (0.78 ~ 2.41)		
Ablation lesions						
Single	79 (45.40)	56 (44.09)				
Multiple	95 (54.60)	71 (55.91)	0.822	1.05 (0.67 ~ 1.67)		
FIGO classification						
FIGO 4-6	160 (91.95)	104 (81.89)				
FIGO 0-3	14 (8.05)	23 (18.11)	**0.010**	2.53 (1.24 ~ 5.13)	0.301	1.53 (0.68 ~ 3.42)

Supplementary Table S2 Risk factors for Changes of blood pressure or heart rate in UFs

Variables	*no* Changes of blood pressure or heart rate *(n = 246)*	Changes of blood pressure or heart rate*(n = 55)*	*P*	OR (95%CI)
Age(year)	45.00 (41.00, 47.00)	43.00 (41.00, 48.00)	0.142	0.64 (0.36 ~ 1.16)
Ablation time (s)	738.00 (513.25, 1113.75)	809.00 (559.50, 1107.50)	0.310	1.36 (0.75 ~ 2.44)
Volume(ml)	156.25 (92.83, 253.79)	166.09 (105.86, 326.89)	0.473	1.24 (0.69 ~ 2.23)
TPV(s/ml)	5.76 (4.22, 8.17)	5.98 (4.13, 6.96)	0.473	1.24 (0.69 ~ 2.23)
NPV(ml)	84.70 (52.90, 156.72)	106.00 (68.55, 185.60)	0.268	1.44 (0.75 ~ 2.75)
tSSS				
0 -18	50 (23.58)	14 (26.42)		
18-25	50 (23.58)	12 (22.64)	0.727	0.86 (0.36 ~ 2.04)
25-40	52 (24.53)	13 (24.53)	0.794	0.89 (0.38 ~ 2.09)
>40	60 (28.30)	14 (26.42)	0.667	0.83 (0.36 ~ 1.91)
Pain during MWA				
Mild	201 (81.71)	38 (69.09)		
Moderate-to-Severe	45 (18.29)	17 (30.91)	**0.039**	2.00 (1.04 ~ 3.85)
Ablation lesions				
Single	112 (45.53)	23 (41.82)		
Multiple	134 (54.47)	32 (58.18)	0.617	1.16 (0.64 ~ 2.10)
FIGO classification				
FIGO 4-6	213 (86.59)	51 (92.73)		
FIGO 0-3	33 (13.41)	4 (7.27)	0.217	0.51 (0.17 ~ 1.49)

Supplementary Table S3 Comparison of peripheral blood test indexes before and after MWA

**Increased Indicators**				
	Before MWA	After MWA	P	Abnormally elevated
AST(IU/L)	19.26±0.4227	24.77±0.5768	< 0.001	21（10.00%）
AST/ALT	1.306±0.0283	1.979±0.0763	< 0.001	13（6.19%）
CREA(umol/L)	55.99±0.5917	57.51±0.8345	0.0290	1（0.476%）
TBIL(umol/L)	10.57±0.3329	13.88±0.4715	< 0.001	32（15.24%）
DBIL(umol/L)	1.785±0.0559	2.320±0.0757	< 0.001	0（0.000%）
IDBIL(umol/L)	8.780±0.2821	11.56±0.4022	< 0.001	31（14.76%）
**Decreased Indicators**				
	Before MWA	After MWA	P	decreased by more than 10%
ALB (g/L)	42.03±0.1619	37.49±0.1678	< 0.001	89（42.38%）
HGB (g/L)	111.0±1.607	106.3±1.56	< 0.001	32（15.24%）
RBC (10^12/L)	4.083±0.0296	3.935±0.0292	< 0.001	28（13.33%）
